# Supplementary material for: Cancer cell-intrinsic biosynthesis of itaconate promotes tumor immunogenicity
Source: EMBO J. 2024 Sep 30;43(22):5530–47. doi: 10.1038/s44318-024-00217-y (PMC11574104; doi:10.1038/s44318-024-00217-y)
Supplement: Supplementary file 1 — Appendix [file 44318_2024_217_MOESM1_ESM.pdf]

Appendix for

Cancer cell-intrinsic biosynthesis of itaconate promotes tumor immunogenicity

Zining Wang[1,3], Lei Cui[1,3], Yanxun Lin[1], Bitao Huo[1], Hongxia Zhang[1], Chunyuan Xie[1], Huanling Zhang[1], Yongxiang Liu[1], Huan Jin[1], Hui Guo[1], Mengyun Li[1], Xiaojuan Wang[1], Penghui Zhou[1], Peng Huang[1,2], Jinyun Liu[1,2], Xiaojun Xia[1,*]

1 State Key Laboratory of Oncology in South China, Collaborative Innovation Center for Cancer Medicine, Sun Yat-sen University Cancer Center, Guangzhou, China.

2 Metabolic Innovation Center, Zhongshan School of Medicine, Sun Yat-sen University, Guangzhou, China.

3 These authors contributed equally

*Corresponding author. Email: xiaxj@sysucc.org.cn

Table of contents Page

–Appendix Figure S1 to S10 2-16

–Appendix Table S1 17

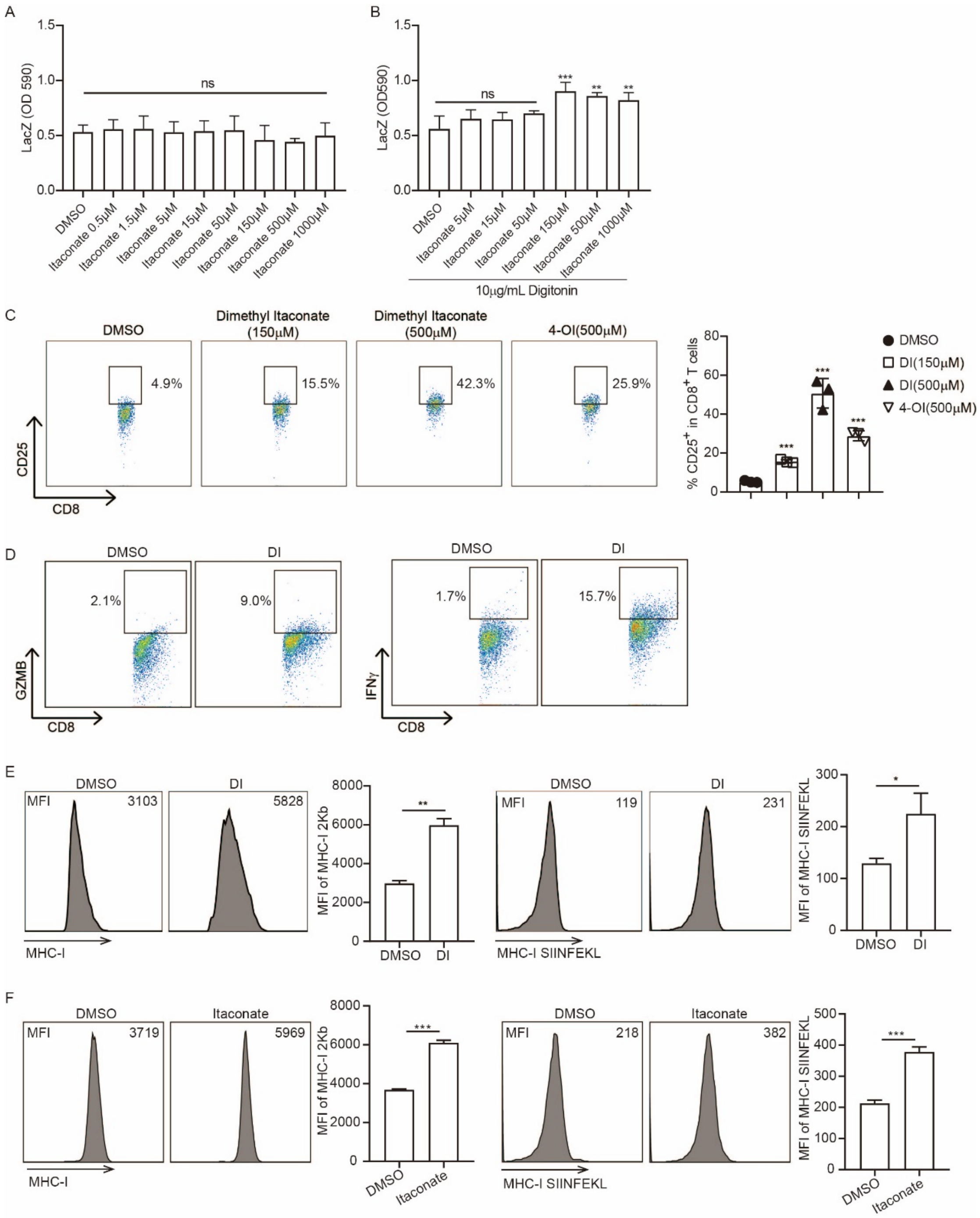

Appendix Figure S1. DI triggers tumor immunogenicity.

A-B. EG7 cells were treated with itaconate or 4-OI directly (A) or itaconate plus digitonin (B) for 16 hrs, then co-culture with B3Z cells for an additional 24 hrs, and then the LacZ activity was measured.

C-D. EG7 cells were treated with DI or 4-OI for 16 hrs, and then co-culture with OT-I for an additional 24 hrs, the CD25 expression and intracellular expression levels of GZMB and IFNγ in T cells were measured by FACS.

E. EG7 cells were treated with DI (500 μM) for 16 hrs, and then the surface expression of MHC-I and MHC-I/SIINFKEL complex was detected by FACS.
F. EG7 cells were treated with itaconate (500 μM) or itaconate plus digitonin (10μg/mL) for 16 hrs, then the surface expression of MHC-I and MHC-I/SIINFKEL complex was detected by FACS.
The graph is shown as mean ± SD of *n* = 3 for all panels. ***p<0.001, **p<0.01, *p<0.05, by unpaired Student's *t* test (E-F) or one-way ANOVA analysis of variance with Bonferroni's post-test (A-C).

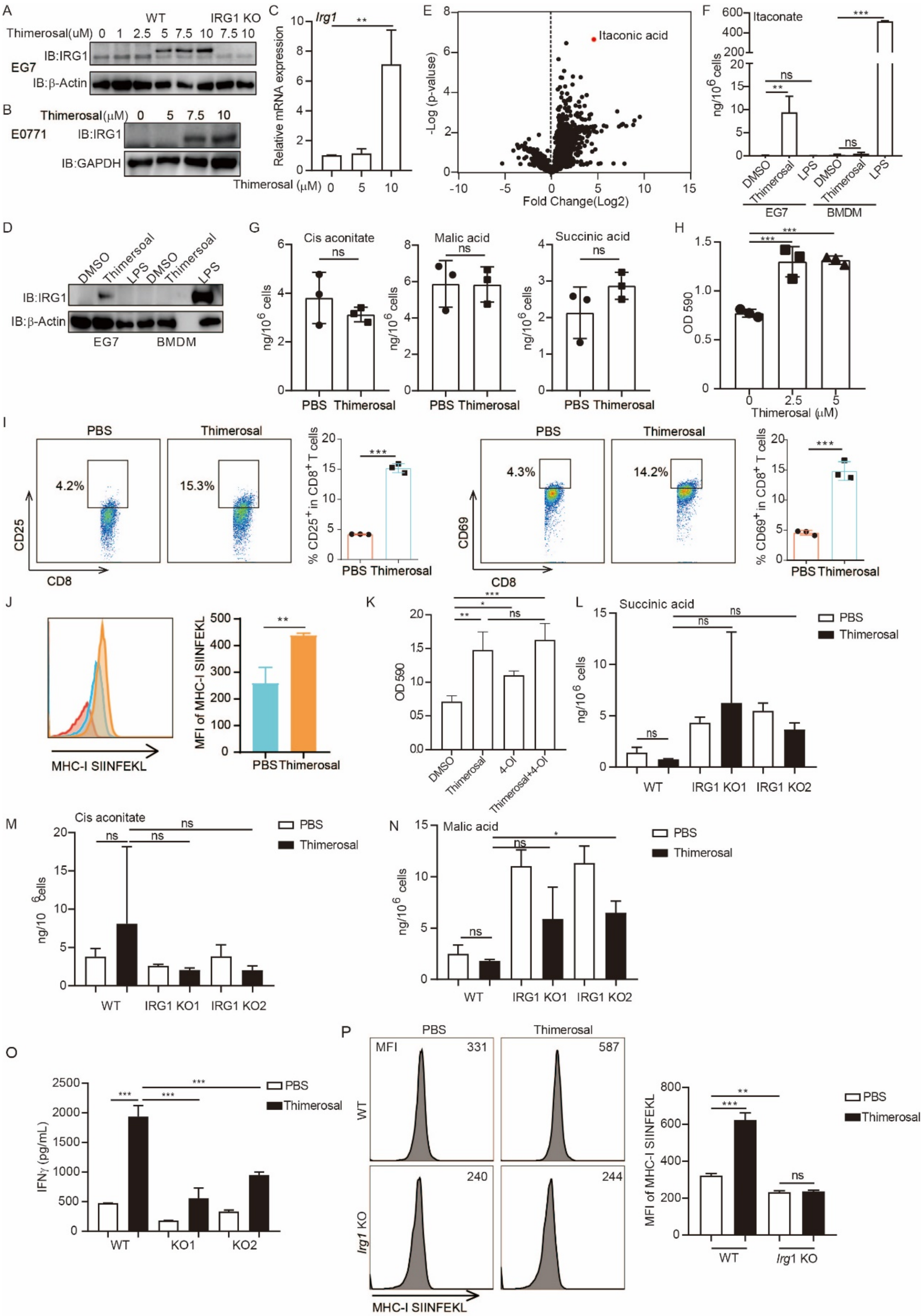

Appendix Figure S2. Thimerosal is an itaconate inducer.

A. EG7 WT or *Irg1* KO cells were treated with indicated thimerosal for 18 hrs, and then the expression of IRG1 was detected by WB.

B-C. E0771 cells were treated with thimerosal or PBS for 18 hrs, and then the expression of IRG1 was detected by WB and qPCR.

D. EG7 cells or BMDMs were treated with thimerosal (10 μM) or LPS (100ng/mL) for 18 hrs, and the expression of IRG1 was detected by WB.
E. EG7 cells were treated with thimerosal (10 μM) or PBS for 20 hrs, and then the cells were harvested for the metabolomic assay. n=4 for each group.
F. EG7 cells or BMDMs were treated with thimerosal (10 μM) or LPS (100ng/mL) for 24 hrs, the itaconate production was measured by HPLC.
G. EG7 cells were treated with thimerosal (10 μM) or PBS for 18 hrs, and then the intracellular *cis*-aconitate, malic acid, and succinic acid were measured by HPLC.
H. TC-1 cells were transfected with OVA (100 μg/mL) for 4 hrs, then treated with Thimerosal (10 μM) for 16 hrs and then co-cultured with B3Z, and the LacZ activity was measured.
I. EG7 cells were treated with thimerosal (10 μM) for 16 hrs, and then the expression levels of CD25 and CD69 on CD8 T cells were measured by FACS.
J. EG7 cells were treated with thimerosal (10 μM) for 16 hrs, and then the surface expression level of MHC-I/SIINFEKL complex was measured by FACS.
K. EG7 cells were treated with 4OI (250 μM) or combined with thimerosal (10 μM), then co-cultured with B3Z for an additional 24 hrs, the LacZ activity was measured.
L-N. WT or *Irg1* KO EG7 cells were treated with thimerosal (10 μM) for 18 hrs, then the intracellular *cis*-aconitate, malic acid, and succinic acid were measured by HPLC.
O. WT or *Irg1* KO EG7 cells were treated with thimerosal (10 μM) for 16 hrs, followed by co-culture with OT-I for another 24 hrs, and then the supernatant IFNγ levels were measured.
P. WT or *Irg1* KO EG7 cells were treated with thimerosal (10 μM) for 24 hrs, and then the expression levels of MHC-I/SIINFEKL were detected by FACS.
The graph is shown as mean ± SD of $n = 3$ for all panels. ***$p<0.001$, *$p<0.05$, ns=not significant, by unpaired Student's *t* test (G-J) or one-way ANOVA analysis of variance with Bonferroni's post-test (C, H, F, K-P).

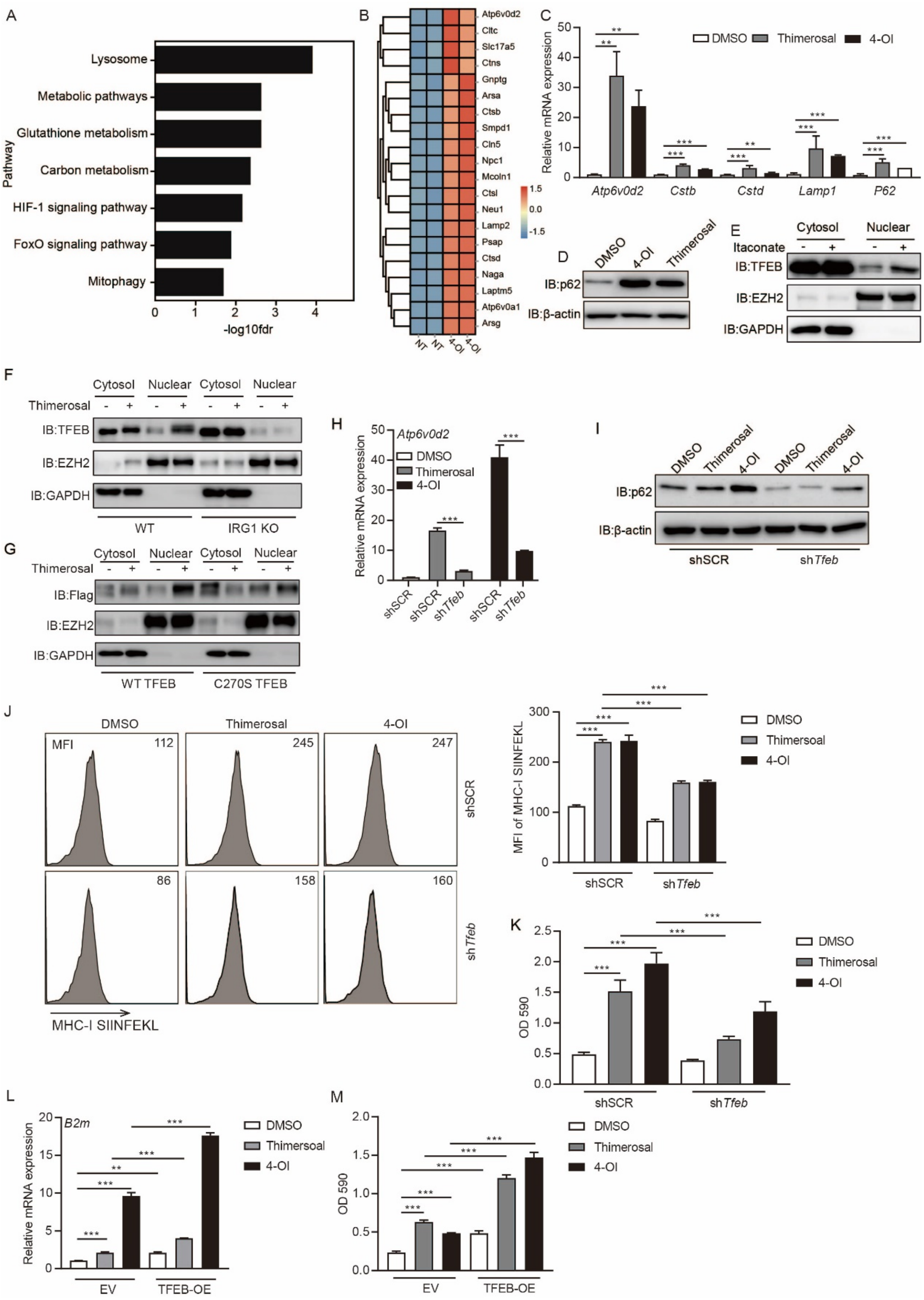

Appendix Figure S3. Itaconate induces tumor immunogenicity by up-regulating antigen presentation via TFEB.

A. Gene set pathway enrichment analysis on RNAseq data using RNA from EG7 cells treated with 4-OI (500 μM) or DMSO for 12 hours.

B. The heat map of expression levels of lysosome biogenesis genes in EG7 cells with 4-OI (500 μM) or DMSO treatment.

C. EG7 cells were treated with thimerosal (10 μM), 4-OI (500 μM) for 16 hrs, and then the lysosome biogenesis genes expression levels were measured by qPCR.
D. EG7 cells were treated with thimerosal or 4-OI (500 μM) for 16 hrs, and the expression of p62 was detected by WB.
E. EG7 cells were treated with itaconate or itaconate (500 μM) plus digitonin (10 μg/mL) for 8 hrs then the expression of TFEB in cytosol and nuclear was detected by WB.
F. WT or IRG1 KO EG7 cells were treated with thimerosal (10 μM) for 18 hrs, then the levels of TFEB protein in cytosol and nuclear fractions were detected by WB.
G. EG7 cells overexpressing Flag-TFEB(WT) or Flag-TFEB(C270S) were treated with thimerosal (10 μM) for 18 hrs, and then the levels of Flag-TFEB protein in cytosol and nuclear fractions were detected by WB.
H. EG7 cells expressing shSCR or sh*Tfeb* were treated with thimerosal (10 μM) or 4-OI (500 μM) for 16 hrs, and then the *Atp6v0d2* expression level was measured by qPCR.
I. EG7 cells expressing shSCR or sh*Tfeb* were treated with 4-OI (500 μM) for 16 hrs, and the expression of p62 was detected by WB.
J. EG7 cells expressing shSCR or sh*Tfeb* were treated with thimerosal (10 μM) or 4-OI (50 μM) for 24 hrs, and then the expression levels of MHC-I/SIINFEKL were detected by FACS.
K. EG7 cells expressing shSCR or sh*Tfeb* were treated with thimerosal (10 μM) or 4-OI (500 μM) for 16 hrs, followed by co-culture with B3Z for another 24 hrs, then the LacZ activity was measured,
L. EG7 cells overexpressing TFEB or empty vector (EV) were treated with thimerosal (10 μM) or 4-OI (500 μM) for 16 hrs, then the *B2m* expression level was measured by qPCR.
M. EG7 cells overexpressing TFEB or empty vector (EV) were treated with thimerosal (10 μM) or 4-OI (500 μM) for 16 hrs, followed by co-culture with B3Z for another 24 hrs, then the LacZ activity was measured,
The graph is shown as mean ± SD of *n* = 3 for all panels. **p<0.01, ***p<0.001, by one-way ANOVA analysis of variance with Bonferroni's post-test (C, H, J-M).

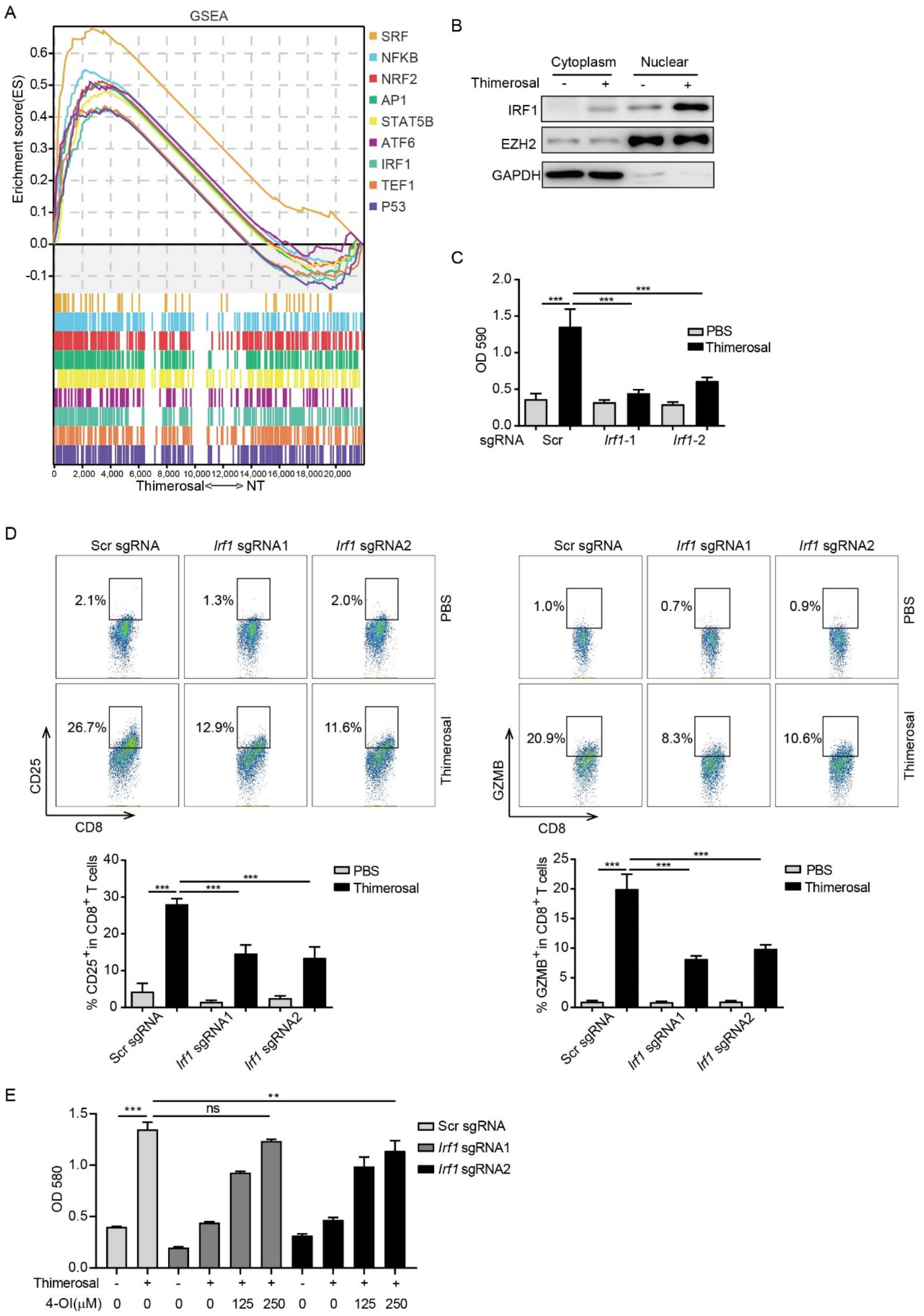

Appendix Figure S4. Thimerosal induces Irg1 expression and itaconate production via IRF1.

A. GSEA analysis of up-regulated transcription factors by thimerosal treatment.

B. TC-1 cells were treated with thimerosal (5 μM) for 8 hrs, then the expression level of IRF1 in nuclear and cytosol was detected by WB.

C. EG7 cells expressing sgSCR or sg*Irf1* were treated with thimerosal (10 μM) for 16 hrs, and then co-cultured with B3Z for an additional 24 hrs, and LacZ activity was measured.
D. EG7 cells expressing sgSCR or sg*Irf1* were treated with thimerosal (10 μM) for 16hrs, and then co-cultured with OT-I for an additional 24 hrs; the expression of GZMB and CD25 in OT-I cells was detected by FACS.
E. EG7 cells expressing sgSCR or sg*Irf1* were treated with thimerosal (10 μM) or combined with 4-OI (250 μM) for 16 hrs, followed by co-culture with B3Z for another 24 hrs, and then the LacZ activity was measured.
***p<0.001, **p<0.01, ns=not significant, by one-way ANOVA analysis of variance with Bonferroni's post-test (C-E).

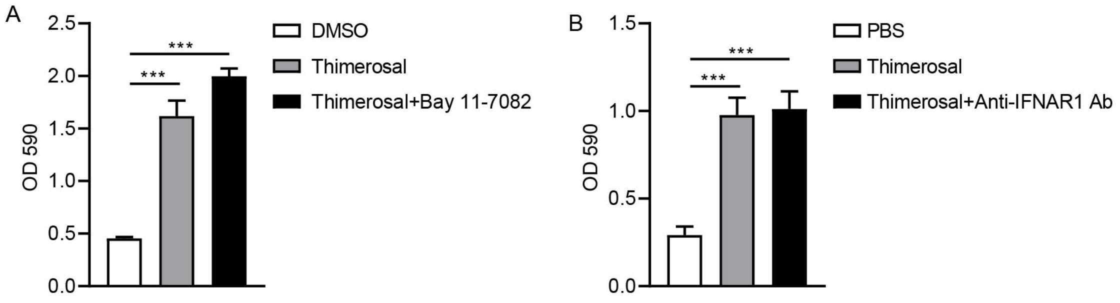

Appendix Figure S5. Thimerosal induces tumor immunogenicity independent of NF-κB and IFNAR1 signaling.
A-B. EG7 cells were treated with thimerosal (10 μM) together with or without Bay 11-7082 (5μM) or anti-IFNAR antibody (Ab) (5 μg/mL) for 16 hrs, then co-cultured with B3Z for an additional 24 hrs, and then the LacZ activity was measured.
The graph is shown as mean ± SD of *n* = 3 for all panels. ***p<0.001, by one-way analysis of variance with Bonferroni's post-test.

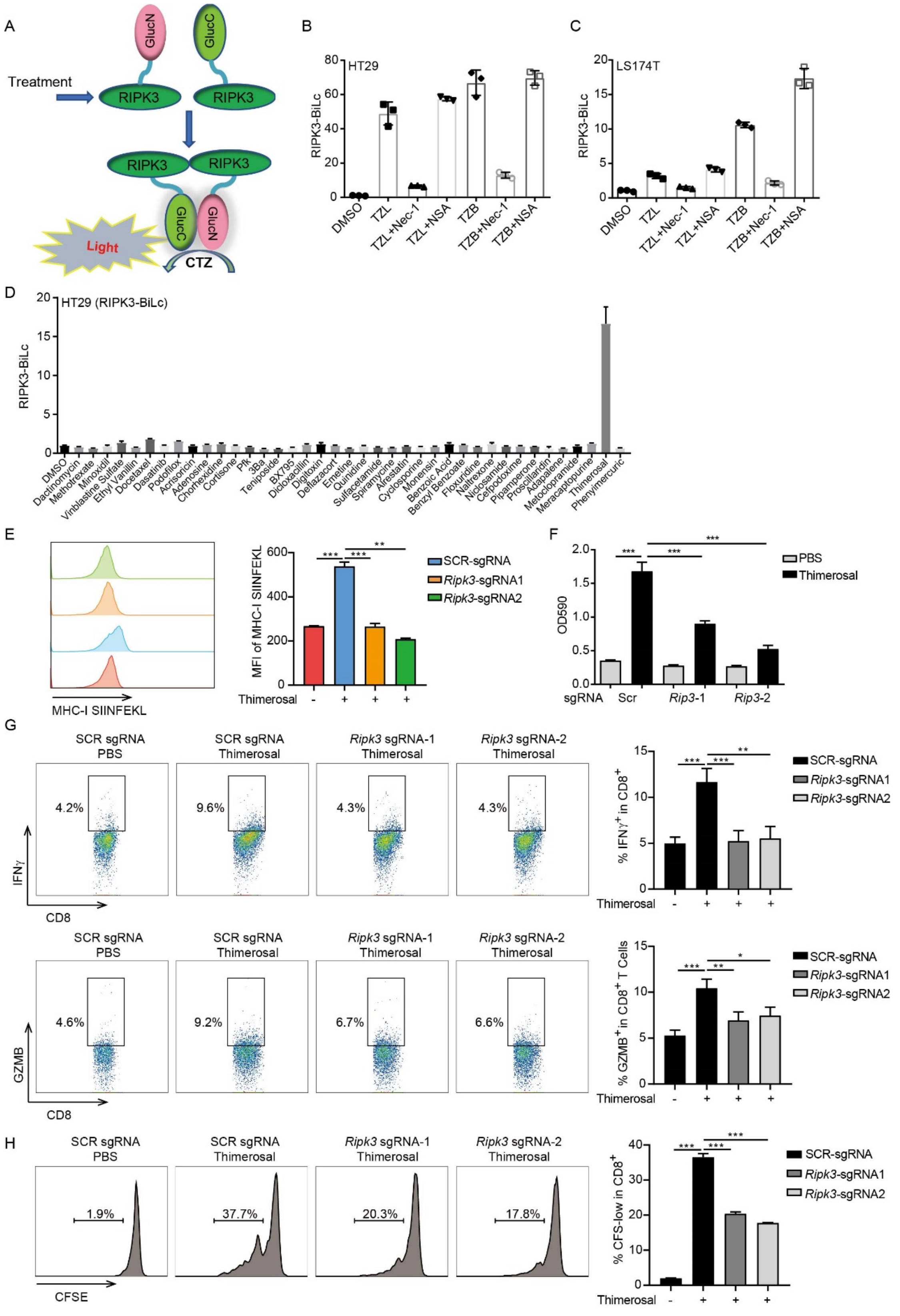

Appendix Figure S6. RIPK3 is essential for thimerosal-induced IRG1 expression and tumor immunogenicity.

A. A cartoon illustration of RIPK3-BiLC reporter used for monitoring RIPK3 dimerization.

B-C. HT29 or LS174T RIPK3-BiLC reporter cells were treated with TZL (TNFα+Z-VAD-

fmk+LCL161) or TZB (TNFα+Z-VAD-fmk+ Birinapant) with or without Nec-1 and NSA, then the luciferase activity was measured.

D. HT29 RIPK3-BiLC reporter cells were treated with potential immunogenicity-inducing drugs (5μM) for 8 hrs, then the luciferase activity was measured.

E. EG7 *Ripk3* sgRNA cell lines were treated with thimerosal (10 μM) for 16 hrs, and then the expression level of MHC-I/SINFEKL was measured by FACS.

F-H. EG7 cell lines were pre-treated with thimerosal (10 μM) for 16 hrs before co-culture with B3Z or OT-I cells for an additional 24 hrs, then the LacZ activity was measured, and the T cell activation and proliferation were measured by FACS.

The graph is shown as mean ± SD of $n = 3$ for all panels. ***p<0.001, **p<0.01, ns=not significant, by one-way ANOVA analysis of variance with Bonferroni's post-test.

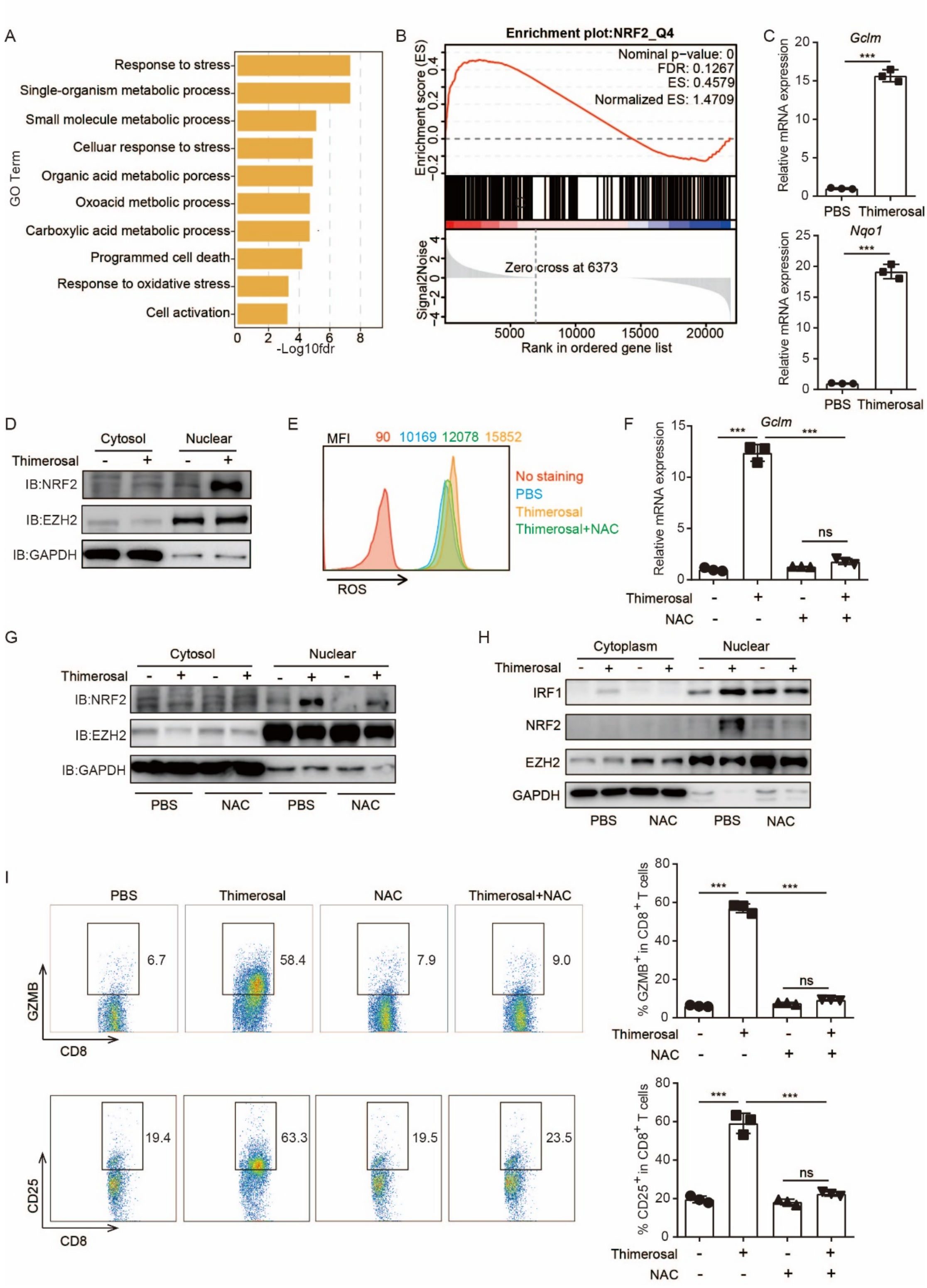

Appendix Figure S7. ROS plays a critical role in thimerosal-induced tumor itaconate production and tumor immunogenicity.

A. Top 10 GO terms enriched in RNA-seq data of thimerosal-treated EG7 cells.

B. GSEA analysis showing NRF2 transcription upregulation in tumor cells by thimerosal.

C. EG7 cells were treated with thimerosal (10 μM) for 12 hrs, and then the expression levels of *Gclm* and *Nqo1* were measured by qPCR.

D. EG7 cells were treated with thimerosal (10 μM) for 8 hrs, and then NRF2 expression in nuclear and cytosol fractions was detected by WB.

E. EG7 cells were treated with thimerosal (10 μM) or in combination with NAC (1mM) for 8 hrs, and then the cellular ROS levels were detected by DCFDA staining followed by FACS analysis.

F. EG7 cells were treated with thimerosal (10 μM) or in combination with NAC (1mM) for 12 hrs, then the expression level of *Gclm* was measured by qPCR;

G. EG7 cells were treated with thimerosal (10 μM) or combined with NAC (1mM) for 8 hrs, and then NRF2 expression in nuclear and cytosol fractions was detected by WB.

H. TC-I cells were treated with thimerosal (10 μM) or combined with NAC (1mM) for 8 hrs, then NRF2 and IRF1 expression in nuclear and cytosol were detected by WB.

I. EG7 cells were treated with thimerosal (10 μM) alone or in combination with NAC (1mM) for 16 hrs, followed by co-culture with OT-I cells for an additional 24 hrs, then the surface expression levels of CD25 and the intracellular expression levels of IFNγ in OT-I cells were measured by FACS.

The graph is shown as mean ± SD of *n* = 3 for all panels. ***p<0.001, ns=not significant, by one-way ANOVA analysis of variance with Bonferroni's post-test.

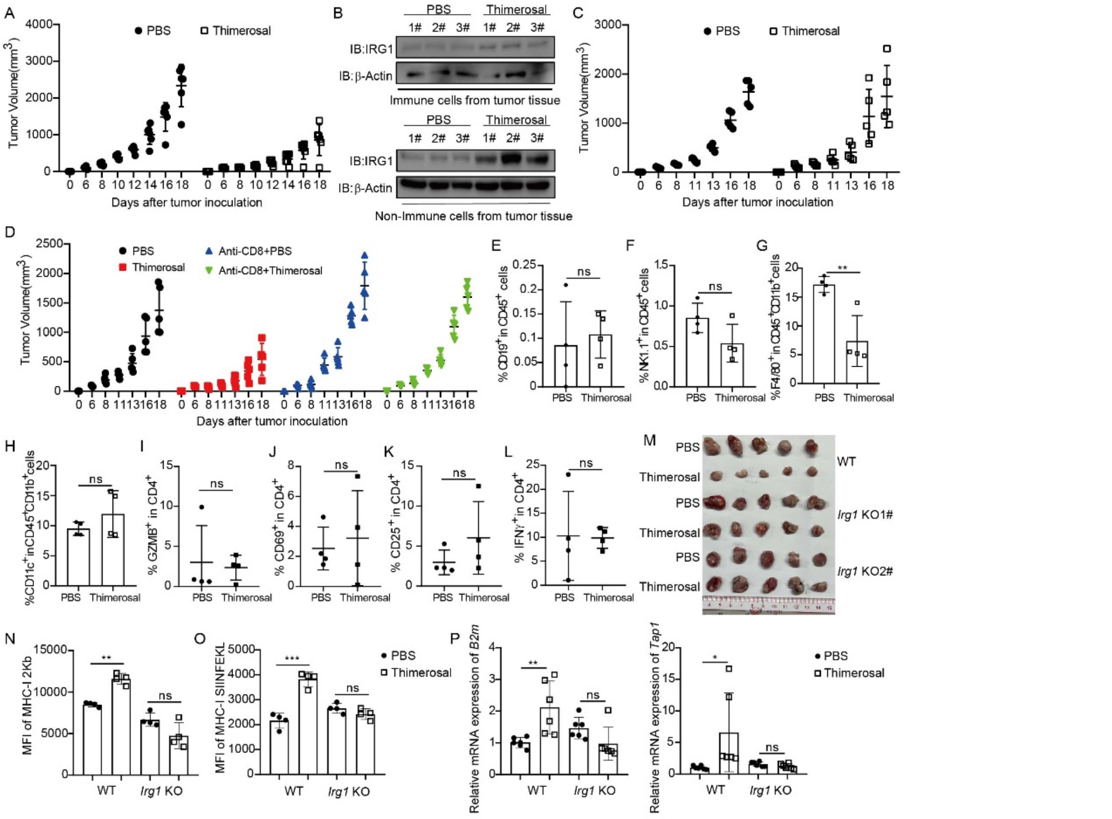

Appendix Figure S8. tumor-intrinsic itaconate induction by thimerosal enhances anti-tumor immunity.

A. Individual tumor growth curve of each B6 mouse bearing EG7 tumor treated with PBS or thimerosal.

B. EG7 tumor-bearing mice were intra-tumoral injected with PBS or thimerosal, then the

tumor tissues were collected after 24 hrs, and made into single cell suspensions used for sorting the immune cells ($CD45^+$) or non-immune cells ($CD45^-$) by flow cytometry. The expression of IRG1 in these cells were detected by WB. n=3 for each group.

C. Individual tumor growth curve of each nude mouse treated with PBS or thimerosal.

D. Individual tumor growth curve of each B6 mouse treated with PBS or thimerosal, with or without anti-CD8 co-treatment. ns=not significant, by unpaired Student's *t* test.

E-L. EG7 tumors were treated with intratumor injection of PBS or thimerosal on day 6 after inoculation, then tumors were harvested on day 10 for immunotyping analysis by FACS.

M. WT or $Irg1^{-/-}$ EG7 cells (1 million per mouse) were subcutaneously (s.c.) inoculated in the flank of B6 mice, followed by intra-tumoral injection of thimerosal or PBS on day 7, then the tumor growth was recorded. The image shows the EG7 tumors on day 15.

N-P. WT or $Irg1^{-/-}$ EG7 cells were subcutaneously (s.c., 1 million cells per mouse) inoculated in the flank of B6 mice, followed by intra-tumoral injection of thimerosal or PBS on day 7, and the tumors were harvested on day 11. The expression of MHC-I or MHC-I/SIINFEKL or immunotyping was analyzed by FACS; and the expression of B2m or Tap1 were detected by qPCR.

The graph is shown as mean ± SD of $n = 4$ for E-L and N-P. ***p<0.001, **p<0.01, *p<0.05, ns=not significant, by one-way ANOVA analysis of variance with Bonferroni's post-test (N-P) or unpaired Student's *t* test (E-L).

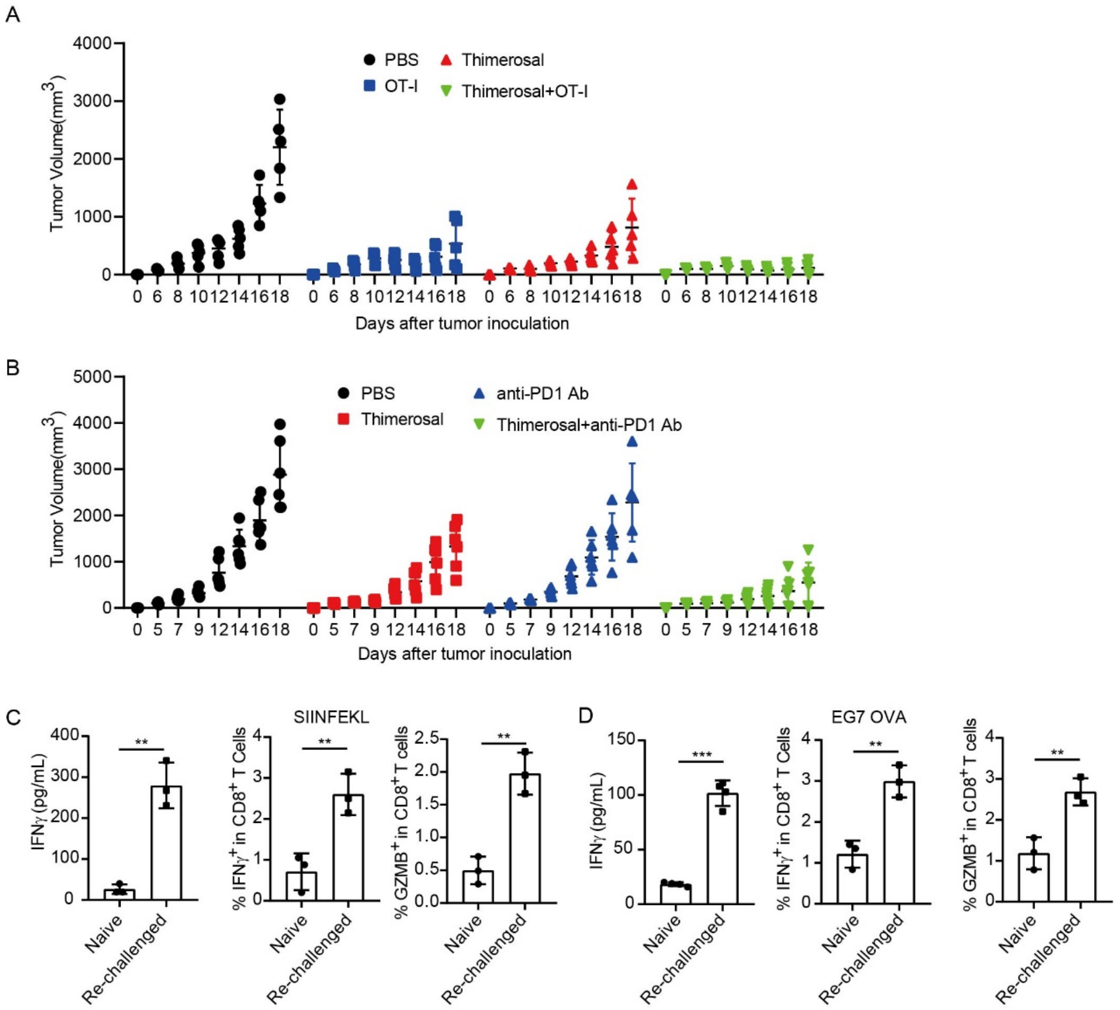

Appendix Figure S9. Tumor-intrinsic itaconate induction by thimerosal sensitizes tumor response to immunotherapy.

A-B. Individual tumor growth curve of each B6 mouse bearing EG7 tumor with indicated treatment.

C-D. Cells were isolated from the lymph nodes of mice that completely rejected EG7 tumors by combination therapy (thimerosal and OT-I), then stimulated with SIINFKEL peptide (2μg/mL) or co-cultured with EG7 cells pre-treated with thimerosal for additional 24 hours; then IFNγ production was measured by ELISA, and the intracellular expression levels of GZMB and IFNγ were measured by FACS.

The graph is shown as mean ± SD of $n = 3$ for C-D. ***p<0.001, **p<0.01, by unpaired Student's t test (C-D).

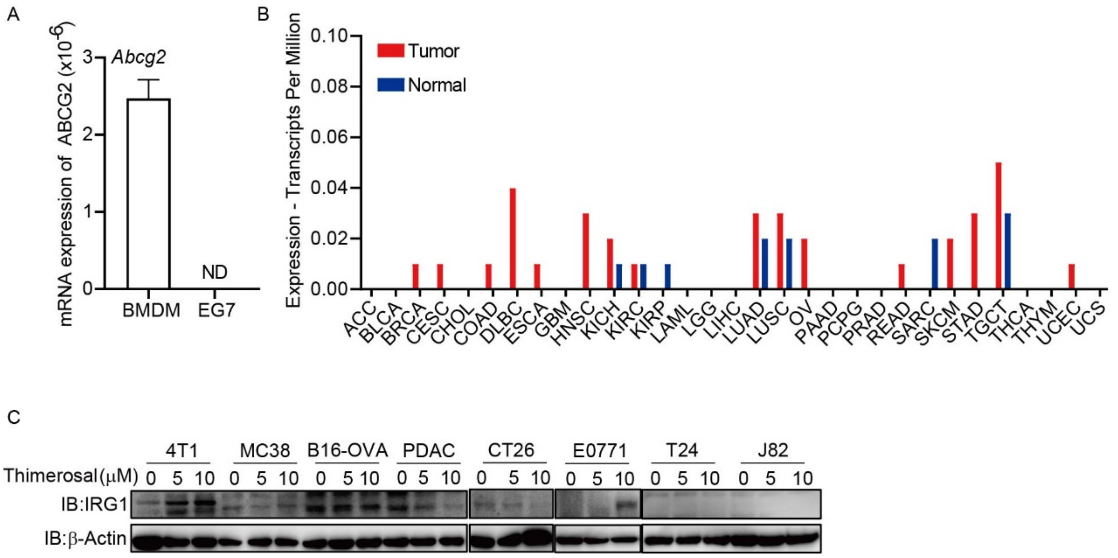

Appendix Figure S10. Low IRG1 expression in human tumor tissues and human/mouse cancer cell lines.

A. The mRNA expression levels of ABCG2 in BMDMs or EG7 cells were detected by qPCR. ND: not detectable. The graph is shown as mean ± SD of $n = 3$ for each panel.

B. The expression of IRG1 mRNA in multiple types of human tumor tissues and normal tissues derived from the GEPIA (Gene Expression Profiling Interactive Analysis) (cancer-pku.cn). ACC: Adrenocortical carcinoma; BLCA: Bladder Urothelial Carcinoma; BRCA: Breast invasive carcinoma; CESC: Cervical squamous cell carcinoma and endocervical adenocarcinoma; CHOL: Cholangio carcinoma; COAD: Colon adenocarcinoma; DLBC: Lymphoid Neoplasm Diffuse Large B-cell Lymphoma; ESCA: Esophageal carcinoma; GBM: Glioblastoma multiforme; HNSC: Head and Neck squamous cell carcinoma; KICH: Kidney Chromophobe; KIRC: Kidney renal clear cell carcinoma; KIRP: Kidney renal papillary cell carcinoma; LAML: Acute Myeloid Leukemia; LGG: Brain Lower Grade Glioma; LIHC: Liver hepatocellular carcinoma; LUAD: Lung adenocarcinoma; LUSC: Lung squamous cell carcinoma; OV: Ovarian serous cystadenocarcinoma; PAAD: Pancreatic adenocarcinoma; PCPG: Pheochromocytoma and Paraganglioma; PRAD: Prostate adenocarcinoma; READ: Rectum adenocarcinoma; SARC: Sarcoma; SKCM: Skin Cutaneous Melanoma; STAD: Stomach adenocarcinoma; TGCT: Testicular Germ Cell Tumors; THCA: Thyroid carcinoma; THYM: Thyroid carcinoma; UCEC: Uterine Corpus Endometrial Carcinoma; UCS: Uterine Carcinosarcoma. C. Human or murine tumor cell lines were treated with thimerosal for 16 hrs, then the IRG1 expression was detected by WB. 4T1: mouse mammary gland tumor cell line from Balb/c background; MC38: mouse colon cancer cell line; B16-OVA: mouse melanoma cell line expressing ovalbumin; PDAC: mouse pancreatic cancer cell line derived from a *K-ras*^{G12D}:*p53*^{R153H} mutant background; CT26: mouse colon cancer cell line; E0771: mouse mammary gland tumor cell line from B6 background; T24 and J82 are human bladder cancer cell lines.

Appendix Table S1 The drugs used for tumor immunogenicity induction screening

Number	Drugs	Number	Drugs
1	Dactinomycin	16	Emetine Dihydrochloride
2	Methotrexate(+/-)	17	Quinidine Gluconate
3	Minoxidil	18	Sulfacetamide
4	Vinblastine Sulfate	19	Spiramycin
5	Ethyl Vanillin	20	Alrestatin
6	Docetaxel	21	Cyclosporine
7	Dasatinib	22	Monensin Sodium
8	Podofilox	23	Benzoic Acid
9	Acrisorcin	24	Benzyl Benzoate
10	Adenosine	25	Floxuridine
11	Chlorhexidine Dihydrochloride	26	Niclosamide
12	Cortisone Acetate	27	Naltrexone Hydrochloride
13	Dicloxacillin Sodium	28	Adapalene
14	Digitoxin	29	Metoclopramide Hydrochloride
15	Deflazacort	30	Mercaptopurine
		#	Thimerosal
